# Predictors of Compulsory Re-admission to Psychiatric Inpatient Care

**DOI:** 10.3389/fpsyt.2019.00120

**Published:** 2019-03-21

**Authors:** Barbara Lay, Wolfram Kawohl, Wulf Rössler

**Affiliations:** ^1^Klinik für Psychiatrie und Psychotherapie, Psychiatrische Dienste Aargau AG, Windisch, Switzerland; ^2^Department of Psychiatry, Psychotherapy and Psychosomatics, University Hospital of Psychiatry Zurich, Zurich, Switzerland; ^3^Department of Psychiatry and Psychotherapy, Charité - Universitätsmedizin Berlin, Berlin, Germany; ^4^Laboratory of Neuroscience (LIM 27), Institute of Psychiatry, University of São Paulo, São Paulo, Brazil

**Keywords:** compulsory psychiatric hospitalisation, severe mental disorders, psychotic disorder, personality disorder, risk factors, prospective study

## Abstract

**Objective:** This prospective study addresses risk factors of compulsory re-admission focusing on the role of the patient's subjective symptom distress and perceived social support, based on comprehensive patient and external (clinicians, study staff) assessments.

**Methods:** Of the baseline sample, 168 (71%) patients with serious mental disorders, who had been compulsorily admitted to psychiatric inpatient care, were followed over 24 months after discharge within the framework of a RCT.

**Results:** During this time 36% had compulsory re-admissions; risk was highest immediately after discharge. Regression models identified a history of previous compulsory hospitalisations and compulsory admission due to endangerment of others as the predictors most strongly associated with the outcome. Patients diagnosed with a psychotic disorder or an emotionally instable or combined personality disorder were most likely to experience compulsory re-hospitalisation, with poor response to treatment further significantly increasing the risk. The patient ratings of subjective symptom distress or perceived social support had no predictive value for compulsory re-admission, and this study did not provide evidence for a significant prognostic relevance of sociodemographic background factors.

**Conclusions:** The present findings suggest that within individual-level variables disease-related factors are essentially the strongest predictors, but including the patients' subjective perspective does not enhance the prediction of compulsory re-hospitalisation. The psychiatric treatment of patients with recurrent and often challenging behavioural problems, at the more severe end of the spectrum of mental disorders, deserves closer attention if the use of compulsory hospitalisation is to be reduced.

## Introduction

A substantial number of patients are compulsorily admitted to psychiatric inpatient care throughout Europe (1–7) and many of them experience repeated compulsory admissions. Data of the Federal Office of Statistics suggest that between 15 and 21% of all psychiatric admissions in Switzerland were compulsory (years 2002–2009; Canton of Zurich: between 23 and 29%) (8).

Compulsory hospitalisation affects an individual's personal interests and autonomy profoundly, thus touching basic human rights, and should be considered only as a measure of last resort for persons who cannot be helped by other means in a less restrictive setting. The comparatively high rates observed in some countries underline the need to scrutinise the use of compulsory measures in psychiatry. This is what has been advocated by professionals, politicians, patients' and human rights organisations for years, campaigning to reduce the number of compulsory psychiatric admissions.

On that account it is important to identify risk factors for compulsory hospitalisation, especially factors which could be addressed proactively by preventive measures or treatment. However, our knowledge of the factors determining the clinical need for compulsory treatment, is still limited. Serious endangerment of self or others is the main prerequisite for compulsory admission to psychiatry in all Western countries, as it is in Switzerland, too. Nevertheless, it is difficult to predict in which cases endangerment of self or others will lead to compulsory hospitalisation. Moreover, in acute psychiatry no specific prognostic tools exist that might help guide decisions regarding post-discharge monitoring, treatment or rehabilitation planning to prevent further compulsory re-hospitalisation.

The preconditions for compulsory admission to psychiatric care are multifaceted, comprising not only a person's current violent or suicidal behaviour, but also aspects of their patient history, treatment motivation, and social and other contextual factors (9–11). Among the patient-related factors known to be associated with increased endangerment of self or others is the type of disorder: high rates of compulsory admission have been reported most consistently for psychotic, schizophrenic or delusional, disorders (12–14), but also for persons with a history of substance abuse (15). Regarding sociodemographic background factors, an increased risk has been repeatedly reported for ethnic minorities (16), in particular non-white or Black people (17). Several studies have found that male gender (14–16) and being unmarried or living alone (12–15, 18) are associated with a higher risk of compulsory hospitalisation. But there are also other studies in which these factors were not confirmed or have been attributed to underlying mediators (14–16, 18, 19).

It is obvious that a comparison of findings across different countries and mental health care systems is difficult, considering that inconsistencies also might in part mirror population composition, configurations of mental health services, as well as professionals' ethics and attitudes (20, 21). Beyond this, research on compulsory hospitalisation has some limitations so far:

- To explore risk factors, psychiatry usually has to recourse to non-experimental designs and most research in this field also rests on cross-sectional data. Lessons that may be learned by retrospectively searching for predictors therefore are almost inevitably limited, revealing correlates rather than “true” risk factors. To assess the incidence of compulsory admissions and risk (or protective) factors prospective studies are necessary. However, only few studies have adopted a longitudinal (cohort) perspective (e.g., Amsterdam Study of Acute Psychiatry (22–24).
- Many analyses focused on specific patient groups, as e.g., (first admitted) subjects with psychosis (25, 26), narrow age categories (25, 27) (adolescents; <50 years old), specific service settings, as e.g., compulsory community treatment (28) or selected countries or areas (14, 26, 29).
- Moreover, many studies are based solely on routinely collected hospital data or retrospective chart reviews (12, 27–29), thus restricting the range of potentially important factors, direct risk factors as well as confounders.
- Studies exploring the subjective perspective of psychiatric patients are scarce and if at all, often adopted a narrow focus on the patients' retrospective view on their involuntary hospitalisation (30–32). It is unclear whether the patients' subjective symptom distress or their perceived social support might contribute to the prediction of further severe crises rendering these patients more likely to experience compulsory re-admissions.

In this situation long-term studies closely monitoring the clinical course of mental patients might help define the risk and guide treatment planning so as to prevent further coercive measures.

We therefore re-analysed data from a prospective clinical trial in which a group of patients with serious mental disorder and compulsory hospitalisation(s) in the past were followed over 24 months after discharge. We used a comprehensive multiaxial assessment (clinicians, study staff, patient ratings) at discharge from the hospital to determine predictors of compulsory re-admission.

Specifically, we address the following questions:

- Do patients' ratings reflecting their subjective view on symptom distress and perceived social support predict compulsory re-admission after discharge from psychiatric inpatient care and
- which are the most important predictors within this multiaxial personal (patient) and external (clinicians/study staff) assessment?

Beyond that, we aimed to find out to which extent the patients' self-ratings of their mental health functioning correspond to clinical staff ratings.

## Materials and Methods

### Sample

The sample for this study is drawn from a randomised trial to evaluate an intervention programme targeting the prevention of compulsory admission to psychiatric inpatient care. Participants were recruited from a naturalistic user sample of inpatient mental health care in four psychiatric hospitals mandated to provide psychiatric care to adults in the Canton of Zurich, Switzerland. Patients aged 18–65 years who had been compulsorily admitted to psychiatric inpatient care at least once during the past 24 months were included in this study. Participation was not limited to a specific mental disorder, but patients diagnosed with an organic mental disorder (ICD-10: F0), mental retardation (F7) or a behavioural syndrome associated with physical factors (F5) were not included. Furthermore, individuals who could not be contacted by telephone and those with insufficient language skills were not eligible for inclusion either.

### Procedure and Clinical Assessments

After having given informed consent, patients were randomised to the intervention group or a treatment as usual (TAU) comparison group. The intervention programme is described in detail elsewhere (33). In brief, it consisted of: (a) individualised psycho-education focusing on behaviours prior to and during an illness-related crisis, (b) working out a crisis card with the patient and, after discharge from psychiatric inpatient care, (c) a 24-month preventive monitoring based on an individualised checklist. This checklist covered the personal risk factors for relapse (e.g., familial, work or financial problems), personal and social resources as well as information on treatment-related behaviour and use of mental health care services.

Baseline assessment included retrospective data on the patient's history, current psychopathology, individual risk factors and protective factors for further compulsory readmission. Baseline interviews were carried out during a participant's inpatient stay (generally over several sessions), before discharge from the hospital. After discharge from the hospital, mental health care use was assessed in regular telephone contacts. Twelve and 24 months after baseline a comprehensive follow-up assessment was carried out again by means of face-to-face interviews. Interviews were conducted by the members of the study staff, all of them graduated clinical psychologists.

### Measures

Clinical diagnoses as well as data on sociodemographic status, occupational and living situation were retrieved from the patients' medical files. Psychiatric diagnoses were made by the hospital physicians in charge at the participating study centres.

Patients' file data on social background and patients' history were supplemented by information obtained from a structured patient interview. We used the German adaptation of the Client Sociodemographic and Service Receipt Inventory CSSRI-EU (34, 35) to assess detailed information about patients' lifetime service utilisation. If a patient's statement conflicted with information in the patient's file ambiguities were clarified during the baseline assessment. In the same way, mental health care use was determined prospectively by retrieving care-related data from the patients' files (review of medical records over the entire study period) and by information from the study participants using the CSSRI-EU. Thus, the frequency and duration of voluntary and compulsory psychiatric inpatient care episodes (and psychiatric outpatient care) were determined.

The Global Assessment of Functioning Scale GAF of the DSM-IV (36) was applied to assess the patient's global level of psychological, social and occupational functioning. The GAF measures how much a person's symptoms affect his or her daily life on a scale ranging from 1 (severely impaired) to 100 (extremely high functioning).

Moreover, the baseline interviews covered specific problem areas which were considered important for the further course of the disorder, as they might relate to symptom aggravation and compulsory admission. These items were rated using all available information from the participant and (responsiveness to treatment) from the medical files. Ratings were dichotomised (1 = severe problems; 0 = no or only minor problems in this area), “severe problem behaviour” being operationalised as follows:

*Partner relationship*: Unstable, very conflictual relationship (including severe or continued violence); or rapidly changing partnerships; or age >30 y and no permanent relationship to date.

*Working*: Severe or continued problems at work; or person (capable of work) refuses to apply for a job; or left employment or was fired within short periods of time. For persons unemployable on the regular labour market, rating was based on sheltered employment, occupational therapy or other respective types of occupation.

*Responsiveness to treatment*: Lack of response to current or recent treatment (for whatever reason; includes patients who did not accept the recommended treatment measures or dropped out of medical treatment).

To assess the patient's symptomatic distress the Outcome Questionnaire OQ-45 (37) was applied. This self-report questionnaire is widely used in clinical settings to estimate the patient's current mental health functioning and changes over the course of treatment. It comprises 45 items to be rated on a five-point scale (0 = “never”; 1 = “rarely”; 2 = “sometimes”; 3 = “frequently”; 4 = “almost always”). The scale provides an index of mental health functioning (total score) and three subscale scores: symptomatic distress or subjective discomfort (SD), interpersonal relationships with intimate others (IR), and functioning in social roles such as work, homemaking, and leisure activities (SR).

The patients were also asked to rate their perceived social support. The Berlin Social Support Scales BSSS (38), a battery of self-report questionnaires, was applied to measure (1) perceived available support; this scale refers to the anticipated possibility of receiving emotional (4 items) and instrumental support (4 items) in the future; (2) need for support (4 items) and (3) support-seeking (5 items). Patients rate their agreement with the statements on a 4-point scale (1 = “strongly disagree” to 4 = “strongly agree”).

### Statistical Methods

We analysed the time to the first compulsory re-admission after discharge from psychiatric inpatient care as the main outcome measure. Time to compulsory admission was calculated from the retrieved re-admission dates on an exact monthly basis. Observation time was limited to 24 months, after that observations were censored.

The baseline variables specified in Table 1 were considered as “explanatory” variables. In a first step we examined these variables in a bivariate analysis using Pearson correlations. In order to quantify the impact of clinical and social characteristics of patients on the outcome, we carried out Cox (proportional hazard) regression analyses. To model the relationship with “age” we added a quadratic term to allow for non-linearity. The significance level was fixed at 0.05 (two-tailed) in all tests.

**Table 1:** Sample characteristics and univariate associations between baseline variables and compulsory re-admission within 24 months (Cox regression analyses; *N* = 168).

	***N* (%) or**			
	**Mean ± SD**	**HR**	**95% CI**	***P-value***
Intervention group	75 (44.6)	0.61	0.36−1.03	0.065
TAU group (*reference*)	93 (55.4)			
**Socio-demographic data**				
Age (years)^a^	44.7 ± 11.5	1.12	0.94−1.32	0.205
Sex: female (*reference*)	96 (57.1)			
Male	72 (42.9)	0.60	0.35−1.03	0.065
Living situation: Alone (*reference*)	82 (48.8)			0.550
With child(ren)	12 (7.1)	1.25	0.48−3.22	0.650
With partner/children	40 (23.8)	0.77	0.39−1.51	0.450
With others/unknown	34 (20.2)	1.32	0.71−2.46	0.387
Occupation: Unemployed/home-maker (*reference*)	107 (63.7)			0.121
Sheltered employment	17 (10.1)	1.33	0.62−2.83	0.462
Regular labour market	44 (26.2)	0.55	0.28−1.07	0.079
Swiss national (*reference*)	143 (85.1)			
Foreign national	25 (14.9)	1.19	0.60−2.34	0.616
**Patient history/clinical data**				
Duration of illness (years)	17.6 ± 12.7	1.00	0.98−1.02	0.773
First compulsory admission (*reference*)	66 (39.3)			
Compulsory admission(s) in patient history	102 (60.7)	2.81	1.52−5.20	0.001
Compulsory admission due to:				
Danger to self (reference)	121 (72.0)			
Danger to others	47 (28.0)	2.05	1.23−3.43	0.006
Substance use disorder	33 (19.6)	0.67	0.34−1.42	0.319
Schizophrenia, bipolar disorder, mania	70 (41.7)	1.98	1.19 −3.28	0.008
Personality disorder	21 (12.5)	1.73	0.90−3.33	0.099
Other disorders	44 (26.2)	0.30	0.14 −0.67	0.003
**Global clinical ratings**				
GAF	39.4 ± 10.7	1.00	0.97−1.02	0.750
Relationship-severe problems	20 (12.3)	0.78	0.34−1.81	0.564
Employment-severe problems	70 (42.4)	1.58	0.95−2.63	0.081
Poor response to psychiatric treatment	28 (16.7)	2.07	1.15−3.71	0.015
**Patient ratings**				
OQ-45 Symptom distress	1.53 ± 0.68	0.75	0.51−1.07	0.137
OQ-45 Interpersonal relations	1.45 ± 0.59	1.15	0.75−1.76	0.525
OQ-45 Social role	1.40 ± 0.64	0.90	0.60−1.35	0.621
OQ-45 Total score	1.46 ± 0.56	0.88	0.56−1.38	0.581
BSSS Perceived support	3.06 ± 0.55	0.76	0.48−1.19	0.230
BSSS Need for support	2.61 ± 0.63	0.86	0.58−1.28	0.466
BSSS Support seeking	2.63 ± 0.57	1.13	0.73−1.76	0.580

*TAU, Treatment as usual; SD, Standard deviation; HR, Hazard ratio for being compulsorily re-admitted; CI, confidence interval*.

a *The age model included a quadratic term to allow for non-linearity*.

To identify a set of explanatory variables that contribute significantly to the risk of compulsory re-admission we fitted a Cox regression model using backward stepwise variable selection based on likelihood ratio statistics. As candidate variables we considered covariates with coefficient *P*-values of <0.1 in the bivariate regression analyses. Moreover, we checked whether an extended Cox-model including a time-dependent intervention effect fitted the data. Since the effect of this time-varying covariate was statistically not significant, it was not further considered in our regression models.

To compare the frequency distribution of the “explanatory” variables included in the Cox regression models (Table 3) between the two treatment groups in the follow-up sample (*n* = 168) we performed Chi-square tests using exact significance levels.

**Figure 1:**
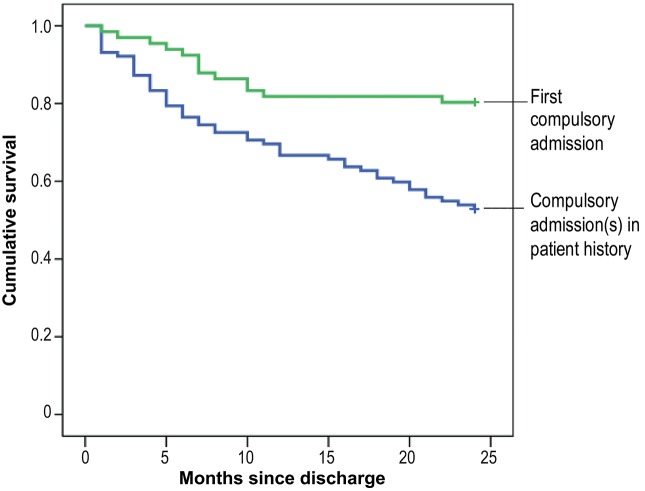
Cumulative risk of compulsory re-admission among patients with vs. those without compulsory admission(s) in their patient history.

**Figure 2:**
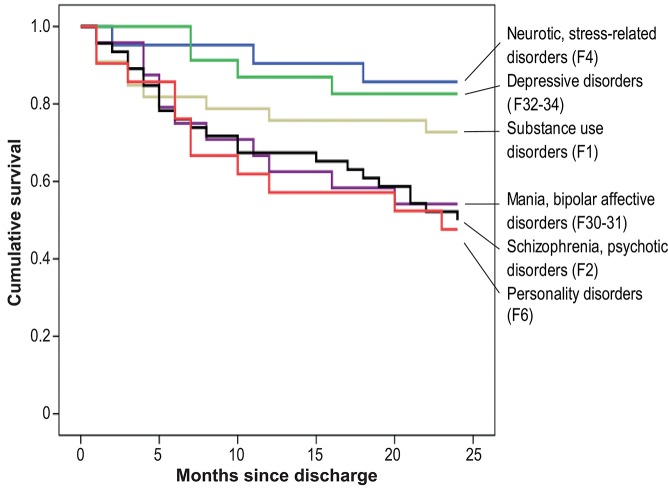
Cumulative risk of compulsory re-admission, by psychiatric diagnosis.

We computed Kaplan-Meier product limit estimates of survival to illustrate the effects of particular significant predictors. The survival curves displaying the estimated survival probabilities (estimated percentages of subjects not compulsorily re-admitted after discharge from psychiatric inpatient care) thus are compared for subjects with vs. those without compulsory admissions in their patient history (Figure 1) and for different diagnostic groups (Figure 2). Statistical analyses were carried out using SPSS 25.

## Results

### Sample Characteristics

Of the 238 participants included in this study, 168 (70.6% of the baseline sample) remained in the study up to the 24 month follow-up. Table 1 provides the baseline sample characteristics of the 168 participants with follow-up assessments over 24 months. The participants suffered from a broad range of mental diseases, of which psychotic disorders were most prevalent: 46 were diagnosed with a schizophrenic disorder (ICD-10: F2), 24 with a mania or bipolar disorder (F30; F31). Across all diagnostic groups psychiatric comorbidity was common and most of the participants showed serious and /or persistent behaviour problems. For the majority of this sample (60.7%) it was not the first compulsory admission, and roughly one in three participant (54; 32.1%) had already experienced four or more compulsory admissions to psychiatric inpatient care in the past.

Regarding their sociodemographic background the sample (mean age: 45 years; 56.0% between 35 and 55 years) is characterised by a high rate of participants living alone and not employed on the regular labour market.

Corresponding to the severity of the disorders, the level of functional impairments was high: according to the Global Assessment of Functioning (staff ratings) the patients showed major impairment in several areas, such as work or school, family relations, judgment, thinking, or mood (mean GAF score: 39.4 ± 10.7).

OQ-45 mean scale scores (patient ratings) ranged between 1 and 2 in all domains. This suggests that the patients themselves described their current mental health functioning at discharge as “rarely” or “sometimes” experiencing symptomatic distress, or distress with respect to interpersonal relationships or social roles.

According to the Berlin Social Support Scale “perceived support” they perceived some degree of social support (mean score 3.1; equal to “somewhat agree”). Regarding the aspects “need for support” and “support-seeking” (with average scale values of 2.6) the patients' ratings are in the middle of the scale, ranging between “disagree” and “agree.”

### Relationship Between Baseline Measures

Pearson's correlation coefficients indicate high correlations between all OQ-45 measures (subscale scores SD, IR, SR, and OQ-total) and moderate to high correlations between the BSSS subscale scores (Table 2). Likewise, the (staff) Global Assessment of Functioning was consistent with the staff ratings of specific problem areas (significant negative correlations). Low level of functioning (GAF), e.g., was significantly associated in particular with severe or continued problems at work, but also with inadequate response to treatment.

**Table 2:** Relationship between ratings by patients, ratings by study staff and patient characteristics at baseline.

		**Age**	**Duration of illness**	**OQ**	**OQ**	**OQ**	**OQ**	**BSSS**	**BSSS**	**BSSS**	**GAF**	**Relation-ships problems**	**Employ-ment problems**	**Response to treatment**
				**SD**	**IR**	**SR**	**total**	**PS**	**NS**	**SS**				
Age	*r*	1	0.382	−0.106	−0.031	−0.086	−0.086	−0.095	−0.161	−0.179	0.083	−0.039	−0.041	−0.006
	*P*		0.000	0.173	0.693	0.272	0.270	0.226	0.038	0.021	0.283	0.623	0.600	0.935
Duration of illness	*r*		1	0.106	0.182	0.108	0.148	−0.040	−0.047	−0.027	−0.220	−0.012	0.078	−0.075
	*P*			0.177	0.020	0.172	0.060	0.615	0.551	0.731	0.005	0.882	0.325	0.338
OQ SD	*r*			1	0.670	0.713	0.908	−0.284	0.267	−0.122	−0.217	0.175	0.182	0.168
	*P*				0.000	0.000	0.000	0.000	0.001	0.119	0.005	0.026	0.020	0.031
OQ IR	*r*				1	0.617	0.855	−0.414	0.082	−0.219	−0.067	0.134	0.132	0.165
	*P*					0.000	0.000	0.000	0.295	0.005	0.390	0.090	0.094	0.034
OQ SR	*r*					1	0.883	−0.238	0.195	−0.054	−0.149	0.117	0.206	0.138
	*P*						0.000	0.002	0.012	0.489	0.056	0.140	0.008	0.076
OQ total	*r*						1	−0.349	0.210	−0.146	−0.167	0.162	0.198	0.178
	*P*							0.000	0.007	0.062	0.031	0.040	0.011	0.022
BSSS PS	*r*							1	0.183	0.454	0.085	−0.189	−0.113	−0.143
	*P*								0.018	0.000	0.277	0.017	0.152	0.065
BSSS NS	*r*								1	0.531	−0.033	0.066	0.042	0.011
	*P*									0.000	0.671	0.407	0.593	0.888
BSSS SS	*r*									1	−0.033	−0.016	0.008	−0.133
	*P*										0.674	0.844	0.921	0.088
GAF	*r*										1	−0.210	−0.303	−0.255
	*P*											0.007	0.000	0.001
Relationships problems	*r*											1	0.232	0.078
	*P*												0.003	0.325
Employment problems	*r*												1	0.363
	*P*													0.000
Response to treatment	*r*													1
	*N*	168	164	166	166	166	166	166	166	166	168	163	165	168

*r, Pearson correlation coefficient; P, P-value*.

Between staff ratings and patients' self-report ratings, however, only limited correspondences were found. The GAF level of functioning showed a significant negative correlation with (OQ-) symptom distress and the OQ-total score, but no significant association was apparent with regard to the other OQ domains (interpersonal relations; social role functioning) or the BSSS ratings (“perceived support”; “need for support”; “support-seeking”).

Remarkably, we found no evidence of a significant correlation between the duration of the illness and the patients' perceived mental health functioning (OQ-45 subscales) or perceived social support (BSSS subscales).

Likewise, there was no indication of sex-specific differences in the perception of social support (BSSS subscale means: no significant differences). There were slight (statistically significant) differences, however, depending on the patients' living situation: the 40 patients who were “living together in a family or with a partner” reported the lowest support-seeking scores (BSSS subscale “support-seeking”: mean 2.44 ± 0.53), whereas the highest scores were found in the single-parent group (mean 2.83 ± 0.65; Living situation: *F* = 3.303; 3 df; *p* = 0.022).

### Compulsory Re-admissions Over 24 Months

During the 24 month follow-up period after discharge from psychiatric inpatient care, 61 of the 168 participants were compulsorily re-admitted to psychiatry: 21 from the intervention group and 40 from the TAU group. A detailed analysis of intervention effects which is not the subject of the present paper is given in Lay et al. (39). In individual cases up to 5 compulsory re-admissions were registered during the 24-month follow-up period.

At 9 compulsory re-admissions within the first month, the number peaked immediately after discharge from psychiatric inpatient care; the likelihood of a first compulsory re-admission then gradually declined over time. The Kaplan-Meier survival curves given in Figures 1, 2 clearly show this risk curve.

### Predicting Compulsory Re-admission

(1) The results of univariate Cox regression analyses revealed that a series of patient characteristics are related to the risk of compulsory re-admission (Table 1). The factors increasing the risk most strongly originated in the patients' history and psychopathology: in particular subjects already with compulsory admissions in their patient history (HR 1.78), with compulsory admissions due to severe danger to others (HR 2.05), the diagnosis of a psychotic disorder (HR 1.98) or a personality disorder (HR 1.73) were at a significantly increased risk of compulsory re-admission.

As to sociodemographic patient characteristics, we did not find statistically significant effects. Nor did the patients' subjective ratings of mental health functioning (OQ-45) or social support (BSSS), predict compulsory re-admission. Among the clinical ratings by the staff, “poor treatment response” was the only significant indicator of an increased risk of compulsory re-admission (HR 2.07).

(2) Results of a multivariate analysis controlling for effects of the intervention showed two significant predictors (Table 3, model 1): “Compulsory admission(s) in the patient history,” suggestive of a 2.48 times higher hazard, as compared to “no previous compulsory admissions,” and “endangerment of others” as compared to “endangerment of self” (1.82 times higher hazard).

**Table 3:** Risk factors for compulsory re-admission within 24 months (Cox regression).

	**Model 1**			**Model 2**		
	**HR**	**95% CI**	***P*-value**	**HR**	**95% CI**	***P*-value**
First compulsory admission (reference)						
Compulsory admission(s) in patient history	2.48	1.32−4.65	0.005			
Compulsory admission due to:						
Danger to self (reference)						
Danger to others	1.82	1.05– 3.15	0.032	1.79	1.01−3.16	0.045
Schizophrenia, bipolar disorder, mania				2.16	1.14−4.09	0.018
Personality disorder				2.55	1.15−5.63	0.021
Poor response to psychiatric treatment				1.93	1.04−3.58	0.037
TAU group (reference)						
Intervention group	0.55	0.32−0.95	0.030	0.56	0.32−0.96	0.036

*TAU, Treatment as usual; HR, Hazard ratio; CI, confidence interval*.

*Model 1: Chi^2^ = 19.225; df 3; P < 0.001; −2Log-Likelihood = 560.518*.

*Model 2: Chi^2^ = 26.383; df 5; P < 0.001; −2Log-Likelihood = 575.761*.

Considering that “compulsory admission(s) in the patient history” is a variable, in itself in need of an explanation, rather than explaining the outcome, we fitted a second regression model, omitting this “proxy” variable in order to bring out deeper-seated factors associated with the outcome. According to this Cox regression model 2 an increased risk of compulsory re-admission is associated in particular with specific mental disorders: the highest hazards were observed for personality disorders (HR 2.55) and psychotic disorders (HR 2.16). Beyond the nature of the mental disorder, poor response to treatment emerged as a further significant predictor (HR 1.93). Moreover, “endangerment of others” (again) was included in the model, suggesting a further risk increase by factor 1.79 given all other variables controlled in the model.

Aside from these patient characteristics, model 1 and model 2 both suggest that participants from the intervention group were less likely to be compulsorily re-admitted than those from the TAU group.

By way of example, the impact of two of the predictors is illustrated by means of the Kaplan-Meier survival curves: Figure 1 compares the Kaplan-Meier plot for patients with a first compulsory admission (baseline assessment) and patients with previous compulsory admissions in their patient history. Figure 2 shows the survival curves for different diagnostic groups, i.e., the proportion “surviving” without further compulsory re-admission in each group.

(3) Our regression models are based on patients who achieved the 24 month follow-up (70.6% of the baseline sample). We lost in this RCT significantly more patients in the intervention group (44; 37.0%) than in the TAU group (26; 21.8%). Therefore, dropout effects could have biased our models. To investigate whether the predictor variables given in Table 3 were differentially affected by sample attrition, we tested whether the frequency distribution of the predictor variables is equally distributed across the two groups.

Results did not show statistically significant differences in any of these variables (First compulsory admission chi^2^ = 0.022, *p* = 1.00; Compulsory admission due to danger to self/others chi^2^ = 1.930, *p* = 0.172; Schizophrenia chi^2^ = 1.790, *p* = 0.209; Personality disorder chi^2^ = 0.582, *p* = 0.488; Poor response to treatment chi^2^ = 0.043, *p* = 1.00; all variables df = 1). This suggests that the different attrition rate in the intervention and the TAU group over 24 months had no significant impact on the distribution of the predictor variables in the regression models.

## Discussion

This study is a prospective long-term follow-up of 168 psychiatric in patients with severe mental illness who already had experienced compulsory admission(s) to psychiatric inpatient care. During the 24 months the study participants were followed after discharge, 36.3% had compulsory re-admissions. The present findings suggest that the risk of compulsory re-hospitalisation is particularly high immediately after discharge from psychiatric inpatient care, then gradually decreases, but is noticeably lower only after 12 months.

To determine risk factors of compulsory re-admission we investigated clinical and social information from the patients' perspective, in addition to standard disease-related and socio-demographic data (assessed by clinicians, study staff).

### Predictors of Compulsory Re-admission

(1) *Clinical measures*. According to our regression models the strongest predictors were “clinical” measures: patients with compulsory psychiatric admissions (already) in their patient history were most likely to experience a compulsory re-admission, in particular those for whom serious endangerment of others, i.e., aggressive, violent behaviour, was the reason for hospitalisation. Regarding the psychiatric diagnosis, patients diagnosed with a personality disorder or a psychotic disorder were at the highest risk. The predictors of the present analysis are largely consistent with previous findings: “A history of involuntary admissions proved to be the only independent predictor of involuntary re-admission” in the prospective follow-up study reported by Setkowsky et al. (23) and van der Post et al. (40). Likewise, functional psychoses (12, 13, 16, 19, 29) and more severe symptoms (15, 16) have been repeatedly reported to increase the risk of compulsory hospitalisation.

Personality disorders, in the present study emotionally unstable (ICD F60.3) or mixed personality disorders (F61.0), however, did not appear to be associated with the incidence of compulsory re-admission in previous research. It is not clear whether this is due to the fact that personality disorders are rarely analysed separately, rather typically subsumed under an “other disorder”-category, or whether they are underdiagnosed in medical charts or whether these studies did not have enough power to prove a statistical significant effect. Not least, it might reflect varying admission decision-making processes as regards the indication of hospitalisation in personality disorders (10).

Nevertheless: there is a problem with “predictors” like “higher number of previous compulsory admissions,” “major mental disorder” or “more severe symptoms,” even if they are indeed well confirmed: Though they are plausible and might be useful for descriptive purposes, they are not free from tautology. Previous hospitalisations, e.g., are exactly the result of a process the prevention of which is at issue. They are limited therefore in terms of explanatory power and practical information.

(2) *Ratings by the study staff*. Among the set of ratings made by the study staff only the rating referring to the “response to treatment” was a significant predictor in the present study: patients rated as non-responsive to the current (inpatient) treatment were more likely to experience a compulsory re-admission after discharge from psychiatric inpatient care. This effect might be attributed to lack of motivation and difficulties relating thereto in treating these people, a factor that has been reported to be directly associated with involuntary admission (10, 15). In this context, however, it also should be taken into account that the diagnoses found to be associated with a significantly increased risk are precisely those regarded as gravely interfering with insight into the illness. In terms of the diagnostic spectrum (as well as their social backgrounds) it appears that the present sample has much in common with “high utilizers” of psychiatric services: persons characterised by comparatively disturbed behaviour, aggression, suicidality, manipulative behaviour, with low social adjustment and limited personal relationships (11). A further point to be considered is the therapeutic alliance, which is well known to be related to various types of outcomes (41). The quality of the therapeutic alliance is likely to play a crucial role in whether a patient refuses to accept the recommended treatment, thereby moderating the non-response-outcome association.

(3) *Patient ratings*. A special focus of our study was on the subjective patient view. In particular, we pursued the question of whether the patient-reported symptom distress (symptomatic distress or subjective discomfort, interpersonal relationships with intimate others, functioning in social roles; measured by the OQ-45) and the perceived social support (perceived available emotional and instrumental support, need for support, support-seeking; BSSS) contribute to the prediction of compulsory re-admission. The underlying idea was that these factors might be associated with further serious crises. None of these measures, however, was found to be linked in any clinically meaningful or statistically significant way to the risk of compulsory readmission.

Regarding the OQ-45 the patient ratings suggested an unproblematic level of mental health functioning. Considering that this assessment was made before discharge from psychiatric inpatient care, a relatively high level of adjustment might not quite be unexpected. The self-reported ratings, however, do not match very well to the assessment by the study staff: the ratings of both interpersonal relationships and social role functioning did not correlate significantly with the respective staff ratings, and only weak associations (statistically significant, but low correlations) were found between symptom distress, OQ total score (patient ratings) and the GAF score (staff rating). Of course, the weak association between self-ratings and clinical ratings does not argue against self- assessments. Rather, it might be explained by different perspectives: the yardstick for the clinician's rating of social and psychological functioning usually ranges between superior functioning and severe impairment. Nonetheless, the patients will make an assessment against the background of their individual biography and (implicitly) compare the current state against how they were doing in the past. Moreover, one should bear in mind that the different instruments used for self-assessment and external ratings basically restrict direct comparisons.

Notwithstanding this, neither the patients' self-ratings nor the clinical staff ratings of functional impairment (GAF as well as the assessment of specific problem areas: partner relationship, working) appear to be useful predictors of compulsory readmission. The present findings, therefore, more likely suggest that the type of the mental disorder and the severity of behavioural problems are the factors decisive as to whether a patient returns to compulsory hospitalisation, rather than the patient's functional (social) impairment.

The second domain the patients had to evaluate were cognitive and behavioural aspects of “perceived social support.” There is compelling evidence that social support is importantly associated with mental health status in various ways (coping with stress, quality of life, mortality risk (42–44). Low social support also has been reported to be a factor that increases the likelihood of emergency compulsory admission (9).

The patients' ratings on the BSSS subscale “support-seeking” corresponded quite understandably to their living situation (alone, with partner, with children, with others). This suggests that the respondents indeed provided a differentiated assessment of their help-seeking behaviour. Even so, the results of the present study did not provide evidence that any of the BSSS domains of perceived social support is associated with the risk of compulsory re-hospitalisation.

The differing results as regards the impact of social support might partly be due to differences in the health and welfare systems in which the studies were embedded and which might carry a different weight (relative to private support) from one country to another. In the present study, e.g., a relatively high number of subjects stated that their only or closest contact person was a “professional.” Besides, a fundamental conceptual difference should be borne in mind: whereas the BSSS subscales measure the *perceived* quality of support, other studies assessed objective social indicators (24) or analysed “social exclusion” from the perspective of a mental health officer (9).

(4) *Sociodemographic patient characteristics* had no further predictive value in the present study. Holding an occupation on the regular labour market showed at least a tendency to provide some protection against compulsory re-hospitalisation (bivariate analysis; statistically not significant). This is in line with findings reported from Norway suggesting that patients who received social benefits, not in paid work, have a higher risk of compulsory admission (15, 16). The role of sociodemographic factors for the risk of compulsory hospitalisation is certainly not straightforward. It is obvious that sociodemographic factors are not independent of disease-related features. Considering that the present study included mostly chronically ill patients, it is therefore plausible that sociodemographic factors such as living situation or occupational integration are only of limited explanatory power. Bearing in mind that the present sample comprised patients from different hospitals responsible for the delivery of acute mental health care services, it is unlikely, however, that the given distribution of sociodemographic characteristics is the result of a sheer sample selection effect.

### Limitations and Strengths

This study has several limitations. Firstly, the sample included in this study is not representative of psychiatry patients in total, insofar as all had already experienced compulsory hospitalisations in their patient history thus representing a selected inpatient sample. Secondly, because the subjects in this study originate from a RCT, the study is not a naturalistic follow-up of psychiatry patients. This is crucial for the interpretation of the frequency of compulsory readmission: seeing that participants were involved in a programme addressing the reduction of compulsory readmission, one must not take re-admission rates to be incidence rates.

A further limitation relates to the analysis, which reflects the outcomes only of those study participants who have remained in this study for 24 months (70.6% of the baseline sample). As with all as-treated analyses, bias might be associated with dropout. In a previous analysis, however, it was shown that type and severity of the mental disorder or the nature of endangerment (of self/of others) at admission were not significantly associated with dropout (39). Moreover, there was no indication of a differential dropout effect in the two treatment groups. It is therefore unlikely that the clinical characteristics, which have been identified as the main risk factors, are artefacts due to attrition effects (irrespective of any accordance with the literature).

Furthermore, the potential risk factors analysed in this study are all on the individual patient-level or the patient's close social environment. Factors on a service-system level which are likely to have a share in the use of compulsory hospitalisation were not investigated. To clarify the contribution of such factors further research adopting a broader perspective is necessary (addressing e.g., organisational characteristics, referral procedures, use of crisis intervention practices).

The strengths of this study are its prospective design, which allows the timely assessment of data, avoiding limitations of retrospective investigations (ambiguous/missing data; recall errors), and its long-term perspective, enabling informative modelling of time to event data. The study sample, recruited from a naturalistic user sample of four psychiatric hospitals and including a broad spectrum of disorders, supports generalisation of findings. Moreover, this study is based on a comprehensive assessment and explicitly considered the subjective patient perspective, personal information that rarely has been studied in previous research.

## Conclusions

The present analysis clearly suggests that on the patient-level, the risk of compulsory re-admission is mainly influenced by disease-related factors. Therefore, no effort should be spared to ensure compliance with treatment and treatment success in this special patient group: subjects with serious mental disorder (in particular, people with psychotic disorders or emotionally unstable personality disorders), recurrent severe behavioural problems (aggression, impulsivity, suicidal behaviour) with compulsory admissions in their patient history. These patients should be closely monitored after discharge from psychiatric inpatient care in order to timely detect early signs of a crisis and to optimise use of services. Aftercare already should be arranged during the inpatient stay, providing patients with a list of available low-threshold services and contact persons in the community in order to take account of the fact that risk of a compulsory re-admission is highest immediately after discharge.

Further research is also clearly needed to study service system aspects that determine referral or crisis intervention procedures, in order to work out promising concepts and investigate the conditions under which coercive admission can be prevented. In addressing such questions, psychiatry should set the focus on the needs of those with the most problematical behaviours at the more severe end of the spectrum of mental disorders.

## Ethics Statement

Ethical approval for the study was obtained from the Ethical Review Board for Clinical Studies of Canton of Zurich, Switzerland, and the study was registered with Current Controlled Trials ISRCTN63162737. All subjects gave written informed consent in accordance with the Declaration of Helsinki.

## Author Contributions

WR and BL designed the study and coordinated the data collection. BL performed the statistical analysis and wrote the first draft of the manuscript. WR and WK contributed to the manuscript revision. All authors read and approved the final manuscript.

### Conflict of Interest Statement

The authors declare that the research was conducted in the absence of any commercial or financial relationships that could be construed as a potential conflict of interest.
